# Development and Dematerialization: An International Study

**DOI:** 10.1371/journal.pone.0070385

**Published:** 2013-10-21

**Authors:** Julia K. Steinberger, Fridolin Krausmann, Michael Getzner, Heinz Schandl, Jim West

**Affiliations:** 1 Institute of Social Ecology, Vienna, Alpen Adria Universitaet Klagefurt-Wien-Graz, Vienna, Austria; 2 Sustainability Research Institute, School of Earth & Environment, University of Leeds, Leeds, United Kingdom; 3 Center of Public Finance and Infrastructure Policy, Vienna University of Technology, Vienna, Austria; 4 Ecosystem Sciences, Commonwealth Scientific and Industrial Research Organisation, Canberra, Australia; University of Florida, United States of America

## Abstract

Economic development and growth depend on growing levels of resource use, and result in environmental impacts from large scale resource extraction and emissions of waste. In this study, we examine the resource dependency of economic activities over the past several decades for a set of countries comprising developing, emerging and mature industrialized economies. Rather than a single universal industrial development pathway, we find a diversity of economic dependencies on material use, made evident through cluster analysis. We conduct tests for relative and absolute decoupling of the economy from material use, and compare these with similar tests for decoupling from carbon emissions, both for single countries and country groupings using panel analysis. We show that, over the longer term, emerging and developing countries tend to have significantly larger material-economic coupling than mature industrialized economies (although this effect may be enhanced by trade patterns), but that the contrary is true for short-term coupling. Moreover, we demonstrate that absolute dematerialization limits economic growth rates, while the successful industrialization of developing countries inevitably requires a strong material component. Alternative development priorities are thus urgently needed both for mature and emerging economies: reducing absolute consumption levels for the former, and avoiding the trap of resource-intensive economic and human development for the latter.

## Introduction

The environmental consequences of international economic growth and development have led to increasingly high profile academic and policy debates, with some protagonists calling for a transition to a “green economy” [1], and others for a complete change in focus away from economic growth [2] [3]. The physical dependency of both developing and industrialized economies on environmental resources, in the form of materials and energy, is a key aspect of this debate. Indeed, if economic development can be somehow decoupled from environmental resources, environmental impacts would be strongly reduced, and the risk of global environmental catastrophes potentially mitigated or averted. Economic activity inevitably entails the use of natural resources, but the scale depends on the structure and technical efficiency of economic processes. As a result, many industrialized countries have instituted policies encouraging such decoupling, for instance by setting targets decreasing the material intensity of economic activity [4]–[6]. At best, however, these targets result in a relative rather than absolute decoupling of the economy and its physical basis, and at worst, they simply reward business-as-usual efficiency improvements [7]. It is thus important to understand the history and past trends of material and energy dependency of economies, as well as to distinguish carefully between the development trajectories of countries which are still emerging as industrialized countries, and those which have long completed their industrial transition.

Researchers have offered several competing theories of environmental dependency of economies in the context of industrialization, development and globalised trade. In early stages of development, agricultural activities dominate, and biomass is the most important resource category [8], [9]. During the process of industrialization, biomass extraction and use remains roughly constant (although the economic and workforce shares of agriculture decline through expansion of other sector and mechanization and intensification respectively), while minerals, including construction materials and metals, and technical energy, including fossil fuels, increase dramatically [10]. The ultimate outcome of global development and trade is disputed: some researchers see strong evidence for convergence in economic dependency on energy [11], which can be related to the theory of technical lock-in of dominant technologies [12], whereas others emphasize that the global division of labor between extraction, manufacturing and consumption activities, shown dramatically by Davis and colleagues [13], acts as a systemic barrier to development [14], and may result in environmental conflicts [15].

Once a country has attained a high level of economic and industrial development, some theories state that a combination of factors could lead to the absolute dematerialization and decarbonization of the economy. These factors are generally described as (i) structural shifts in production and consumption patterns (often known as the transition to a “service economy”, although this transition is disputed [16]), (ii) high technical capacity (leading to more efficient use of resources), (iii) surplus resources to devote to environmental protection [17], [18], and/or (iv) economic preferences for outsourcing labor-intensive (and possibly pollution-intensive or primary commodity-intensive) activities [19]. Together, these phenomena could be expected to lead to an “Environmental Kuznets Curve” or EKC, where environmental impacts first grow, then decrease with income, in the shape of an inverted-U. Past studies have found strong evidence for EKC behavior for certain categories of pollutants, which tend to have in common that their impacts are local and immediate, while their abatement is technically straightforward and low cost [18], [20]. Since materials and energy use, along with carbon emissions, do not fit this characterization, there is no reason to believe that they should be subject to the EKC phenomenon. Past studies have found mixed evidence for an EKC for materials [21]–[23]. The EKC studies for carbon are too numerous to cite here, but also show mixed evidence, along with methodological [24] and accounting issues related to trade [25]. Indeed, consumption-based carbon emissions, which include carbon embodied in traded goods and services, show less evidence for an EKC than territorial emissions, for instance [26]. All consumption-based studies demonstrate that developing countries (non-OECD, non-Annex B) are net exporters of both materials and greenhouse gases to industrialized countries, with traded volumes increasing over time [27], [28].

In this article, we analyze material consumption and carbon emissions from both developing and industrialized countries over almost four decades (1970–2005) to identify commonalities and divergences in economic dependency on environmental resources, and offer robust conclusions concerning the potential for absolute decoupling of economic activities from environmental resources. This study is innovative, since it constitutes the largest study of economic dependency on material use in terms of the number of countries, their diversity in development status, and time span covered. For the first time, we include a simple time trend alongside income dependency terms. We investigate total materials, divided into biomass and minerals & fossil fuels, and conduct a full parallel analysis on carbon emissions. We start by describing our data and methods in Section 2. In Section 3, we present the results of our analysis, starting with a cluster analysis to quantitatively determine the development status of countries in our sample, followed by the country-by-country analysis of economic-material coupling trajectories, and culminating a full panel analysis. In Section 4, we discuss the implications of the findings for our understanding of the coupling between economy and resource use, and in Section 5 we conclude that a shift away from industrial development and towards development focused on human well-being is necessary.

## Materials and Methods

### Data

#### Material use

Economy wide material flow accounts provide information on material extraction, trade and consumption in national economies. For this study we have used domestic material consumption (DMC) as the appropriate indicator. DMC measures apparent consumption and is defined as domestic extraction plus imports minus exports of materials [29], [30].

In recent years material flow data has been compiled for an increasing number of national economies, and the quality of economy wide material flow data has greatly improved. Consistent and comparable information on DMC in time series is now available [30]. For this study we sourced the data required for 39 countries plus the world, from two existing economy-wide MFA databases. The MFA database maintained at the Institute of Social Ecology provided published and unpublished economy-wide MFA data for a set of countries in Europe, North and Latin America, Africa and Asia. Published data from this source includes data for the EU-15 member states [31], the USA [32], Japan [33], India [34], Brazil [35], and the global total [8]. The second database used was the CSIRO and UNEP dataset which covers countries in the Asian-Pacific region, available online at www.csiro.au/AsiaPacificResourceFlows. Previously published work which uses this latter database includes [36], [37]; See File S1 for a list of all included countries and sources. All MFA country time series used in this study were constructed using the same methodological principles, referring to guidelines developed by Eurostat [38] and employing state-of-the-art adaptations of accounting procedures for non-European countries. MFA data are compiled on the basis of national and international statistical sources and standardized procedures to account for extraction of and trade with the main material groups biomass, fossil energy carriers, ores and non-metallic minerals. Adaptations for non European countries, for example, concern the assumptions used to estimate biomass flows not covered in statistical sources such as used crop residues or grazed biomass [39]. Due to the high degree of methodological standardisation and the quality of the underlying primary data the comparability of MFA data both across countries and over time is high, as has been shown by Fischer-Kowalski et al [40].

In our analysis we subdivide DMC into two main material groups: biomass and minerals and fossil fues. Biomass includes harvested crops, crop residues and forage, biomass grazed by livestock, timber and fuel wood. Minerals and fossil fuels include metal ores, non-metallic minerals (including sand and gravel for construction) and fossil energy carriers. These groupings are used as they have been shown to have distinct relationships with population and economic development [7], [8], [10], [41].

#### CO2 emissions

Data on CO_2_ emissions from fossil fuel combustion and cement production was taken from the CDIAC database [42].

#### Gross domestic product (GDP) and population

Data on GDP in constant US Dollars of 2005 and population was taken from the United Nations National Accounts Database [43].

The temporal coverage of the data is 1970–2005, except for the DMC indicators of the EU-15, Argentina and Cuba until 2004, 1980–2003 for Canada, and 1972 onwards for CO2 for Bangladesh and Pakistan.

The use of DMC and GDP as environmental and economic indicators can be criticized. Cleveland and Ruth [44], among others, discuss the weakness of aggregating materials based on weight, rather than economic value or environmental impact, for example. However, DMC is intended to measure bulk materials, not specialized or highly polluting materials like neodymium or plutonium, and any non mass-based weighting system would be open to other criticisms, as well as being impractical to assemble credibly for an international database over many decades. On the economic side, GDP has been thoroughly discredited as indicator of wellbeing, with high profile initiatives calling for alternative measures [45]. However, GDP is an indicator of total economic activity, and this aspect is what we seek to understand in this article. The values of key indicators for the countries in our sample are shown for 2005 in File S1.

### Methods

Our analysis is conducted using three separate quantitative methods: a cluster analysis for identifying country placement in the developing/emerging/industrialized hierarchy; regression analysis on individual countries over time for each material category; and finally a full econometric panel analysis conducted on different samples of countries, for each material category. It is worth noting that we conduct all of these analyses on per capita values, i.e. ‘metabolic rates’ [30] rather than including population separately as a contributing variable. This is done in order to simplify the analysis to quantities which are comparable across countries, and to avoid the issues arising from the interpretation of differing population coefficients in different methods [46]–[48].

#### Country groupings: cluster analysis

We conduct cluster analysis to determine development status groupings of the countries in our sample,based on income and per capita material use, both static values and dynamic growth rates. The clustering is based on the average linkage between all pairs of objects in any two clusters, and standardized Euclidean distances in order to make the dimensions unitless and cover the same range. Further details are available in File S1.

#### Individual country trajectories

Each country in our sample faces a unique set of circumstances and events. It is thus interesting to consider the coupling between material use per capita (the metabolic rate) and economic growth (measured as GDP per capita) of each country over time. This is done by measuring the coefficient of log-linear coupling given by the linear-least-squares regression
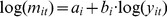
(1)Where 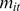 is the metabolic rate (DMC per capita) and 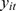 is the income (GDP per capita) of country *i* at time *t*.

The coupling coefficient *b* is of particular significance; it is the *income elasticity of consumption* [49] of a country: in other words it measures the strength and magnitude of the coupling between its economy and resource use. Indeed *b* quantifies the growth in material use per capita for a given growth in income. For example, if *b* = 1, material use per capita will grow exactly proportionally to income, but if *b* = 0.5, material use per capita will only grow at half the rate of income growth. Values of income elasticity between 1 and 0 thus correspond to *relative* decoupling: material use grows with the economy, but not as fast as GDP growth. *Absolute* decoupling occurs when the income elasticity is below 0: then the physical dependency of the economy actually declines with economic growth. It should be noted that this definition of decoupling applies only to GDP and material use per capita, and thus does not take population growth into account: if population grows at an annual rate *p*, the condition for relative decoupling translates roughly to 1>*p+b*>0, and becomes *p+b*<0 for absolute decoupling.

#### Econometric approach and panel analysis

To add to the insights gained by the cluster analysis groupings and material-economy coupling of individual countries, we conduct a full econometric panel analysis of the time series data. In a first step, we test for specific time series attributes: whether the time series follow a stable (stationary) path, or whether they develop according to a unit root process (with stable growth). As our data consists of time series for a broad range of countries, we explore whether the time series follow stationary paths or root unit processes by means of different panel stationarity tests (such as Panel ADF tests). These tests examine whether the time series of a given variable for different countries in the sample follow a common path (e.g. a common trend), or whether they develop independently from one another.

There are several methodological approaches available for panel data such as the one considered here; Stern (2010) presents estimations methods for panel data such as OLS (linear, quadratic, with/without time trend) and compares these to models including fixed and random effects, and between estimates. There are different econometric methodological problems involved with the different approaches, which may lead to differing values of the coefficients. In light of the econometric discussion, we chose the current approach, although it is clear that our results may change with the application of other econometric estimation procedures and models.

From a methodological point of view, simply regressing two non-stationary time series on each other might lead to spurious regressions, indicating that two variables are significantly correlated, while in fact the relationship is non-existent. Thus, the stationarity tests not only produce substantial results regarding the development of the different time series, but are also important for the next steps of the methodological approach.

As a second step, we test for the cointegration of the explanatory as well as dependent variables. Our research goal is to investigate the coupling of resource use (e.g. DMC per capita) and economic development (GDP per capita). We therefore test whether resource use and economic development variables are “cointegrated”, i.e. whether they run parallel (meaning that the residuals of a bilateral (cointegrating) regression are stationary).

If the variables are cointegrated, it can be concluded that they are indeed correlated; for instance, if economic activity and resource use are cointegrated, GDP might very well determine DMC (or vice versa). When estimating this relation, serial correlation (the correlation of a time series variable with itself over time) might constitute a major problem; in order to avoid distorted coefficients, estimations should include the appropriate autoregressive terms to correct for serial correlation.

We expect the correlation between GDP and resource use to follow certain development paths, and the time series to include both long-term trends and dependencies, and short term fluctuations. Due to technological choices, a locked-in path may be mirrored by a rather stable long-term correlation between GDP and resource use, since many technologies cannot be changed in the short term. However, short-term fluctuations in national GDP or industrial production values may certainly lead to coefficients that are very different from the long-term relations. All analyses will also be done for the whole sample (accounting for heteroscedasticity by appropriate weighting), as well as for specific groupings of countries based on development status determined through the cluster analysis. We also test for the presence of a quadratic term (corresponding to Environmental Kuznets Curve behavior if it is negative), and for a simple time-related trend.

As a consequence, the equation used in the panel regressions is as follows:
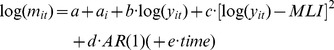
(2)Where *m_it_* and *y_it_* are the material use per capita and GDP per capita, respectively, of country *i* at time *t*, and MLI = mean(log(*y*)) is a constant included to prevent co-linearity between the linear and quadratic income terms [50]. *AR*(1) is the autoregressive term used to correct for serial correlation. We use pooled EGLS (estimated general least squares) with White cross-section standard errors and covariance.

The results of the panel analysis are then the general and country-specific constants *a* and *a_i_*, the linear income coefficient *b* (analogous to the income elasticity of consumption in the individual country analysis described above), the quadratic income coefficient *c*, and if it is included in the analysis, the linear time trend coefficient *e*. In terms of the EKC analysis, the peak income is given by:
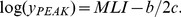
(3)Whether or not the detection of a quadratic term is a proof of delinking (inverted U) or relinking (U) depends on the statistical significance of the coefficient *c*, obtained from the Student T-test. Moreover, the peak income should be within the range of the incomes existing in the sample: otherwise, only one half of the U (i.e. no U at all) has been observed.

## Results and Discussion

### Industrialized, emerging, developing and delayed countries

The main goal of this work is to characterize the differences between mature industrialized countries, which already attained high levels of economic activity and resource use in 1970, at the start of our study period, and countries at other stages in their development: emerging, developing, or simply discontinuous or disrupted. For many countries, the attribution to one group or another is straightforward, but several countries have an intermediate or ambiguous status. We therefore use cluster analysis to define two quantitatively robust country groups.

Traditionally, socio-economic indicators, especially GDP per capita, are used to describe and compare the development status of individual countries. At the core of our research, however, we are interested in the physical and environmental aspects of socio-economic development and industrialization, represented by material consumption per capita (the metabolic rate). When maturing, economies change their resource base which is known as the “metabolic transition” of countries [9], [51]. We thus include material consumption, measured as DMC per capita, alongside economic development, measured as GDP per capita, in our analysis.

The definition of industrialized and developing economies represents a moving target; it changes over time. An income qualifying a country as industrialized in 1970 may not be sufficient in 2004. The status of a country must thus be considered within a specific time period. We thus conduct two separate analyses: at the beginning of the time span of our data, in 1970, and at the end in 2004. This also enables us to detect countries which change status, from developing to industrialized, or vice-versa, over the decades covered in our data.

Because we are interested in development trajectories as well as development status, we define the country clusters in terms of their dynamic (material and economic) growth rates, as well as their material and economic status. From this analysis, which is detailed in File S1, we identify several distinct groups of countries, summarized in Table 1 under three broad categories: “Mature,” “Emerging,” and “Developing.” The “Emerging” category is the most interesting and mixed, ranging from countries which would have been considered industrialized in 1970, but no longer in 2004, to fast developers, with an inverse trend.

**Table 1:** Country types based on cluster analysis.

**1. Mature industrialized (16)**
Austria, Belgium and Luxembourg, Canada, Germany, Denmark, France, Ireland, Italy, Japan, Netherlands, New Zealand, Sweden, United Kingdom, USA
Special cases: Finland and Australia stand out in terms of their high material consumption.
**2. Emerging (17)**
**Successful developers (attained industrialized status by 2000–04):** Greece, Portugal, Republic of Korea, Singapore, Spain
Delayed development (belonged to industrialized group in 1970–75): Argentina, Venezuela
Consistently intermediate: Algeria, Brazil, Cuba, Iran, Malaysia, Turkey
Faster development (belonged to developing group in 1970–75): China, Colombia, Indonesia and Thailand. China stands out in terms of its economic and material growth rates in 2000–2004.
**3. Developing (6)**
Bangladesh, India, Nepal, Philippines, Pakistan, Sri Lanka.

**Figure 1:**
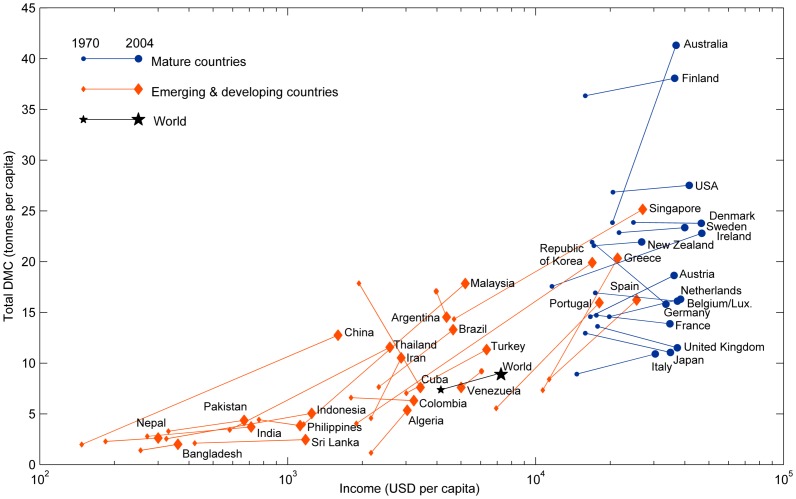
Income and Total Domestic Material Consumption per capita for mature (blue circles), emerging/developing countries (orange diamonds), and the world (black star), in 1970 (small marker) and 2004 (large marker). (Canada is missing data for 1970.)

In the remainder of our analysis, we combine the 2^nd^ and 3^rd^ groups in Table 1 as “Emerging/Developing” countries. Indeed, we expect the economic-material coupling of the “successful developers” and “still emerging” countries to have similarities with the “developing” group, since they theoretically represent stages along the development trajectory. The “Mature” (mostly moving across) and “Emerging/Developing” (mostly moving upwards) groups are shown in terms of their GDP and DMC per capita at the beginning and end of the time series in Figure 1.

We generally expect mature industrialized countries to be distinguished by high levels of economic activity and material consumption, and relatively slow growth rates in both dimensions, while developing and emerging countries would have lower economic and material status, but higher growth rates. Intermediate countries would exhibit various mismatches between their levels of wealth/consumption and growth rates. This is the overall behavior observed in Figure 1. Income plays a larger role than material use in determining the status of a country, as is evident from the clear horizontal separation and significant vertical overlap of the country groups. The results in the cluster analysis (Table 1) and trajectories (Figure 1) both show that there is a great diversity of possible development, in economic and material terms, and that there is not a single dominant trajectory. This diversity will be explored further in terms of economic-material coupling in the next section.

### Economic-material coupling for individual countries

**Figure 2:**
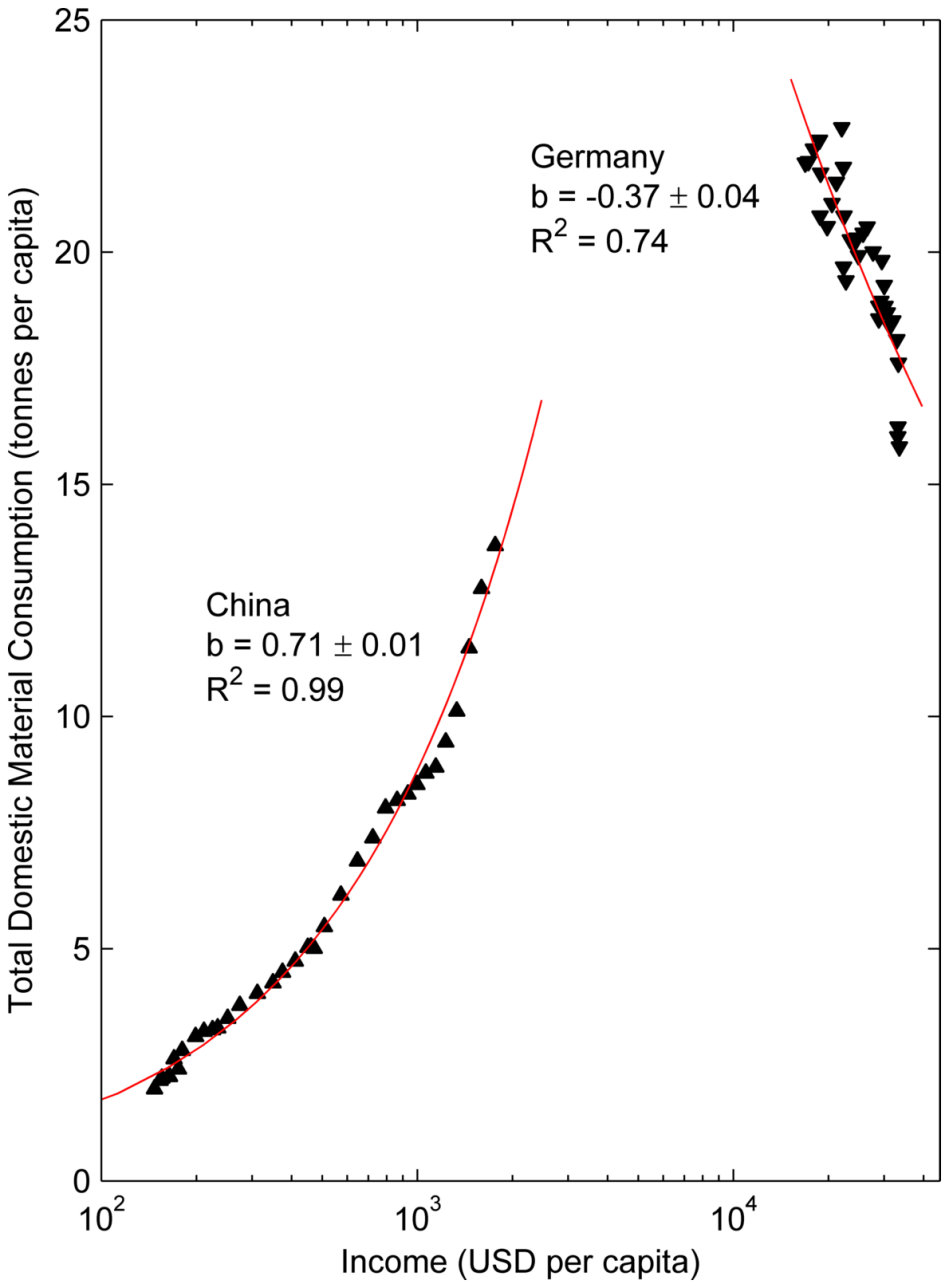
Examples of strong positive economic-material coupling (China, coupling coefficient 0.7), and negative economic-material coupling (Germany, −0.4). The goodness-of-fit R^2^ of the fit curve is also shown.

In this section, we explore the long-term coupling between the economic growth of each country and its material consumption by measuring the income elasticity of material consumption, *b* in Eq. 1, for each country in our sample, over the entire period. Examples of economic-material coupling are shown in Figure 2 for China (strongly positively coupled) and Germany (strongly decoupled). This first analysis shows that individual countries follow very diverse trajectories, which have to be understood in terms of each country's circumstances.

For each country, we thus obtain an economic-material coupling coefficient quantifying the level of material dependency of its economic growth. This is done for total materials, as well as the mineral/fossil and biomass components separately, and compared with values for CO_2_ per capita. The results are summarized in Table 2.

**Table 2:** Coupling coefficients of income and material consumption.

Country group	Country	Total DMC/cap		Mineral and Fossil DMC/cap		Biomass DMC/cap		CO_2_/cap	
**Emerging/**	**Algeria**	2.4	(.31)	2.7	(.36)	1.2	(.21)	2.0	(.32)
**Developing**	**Argentina**	0.4	(.20)	1.7	(.15)	0.1	(.27)	0.5	(.07)
	**Bangladesh**	0.9	(.08)	5.1	(.54)	0.0	(.05)	2.2	(.13)
	**Brazil**	0.9	(.05)	1.6	(.09)	0.6	(.05)	0.9	(.09)
	**China**	0.7	(.01)	0.9	(.03)	0.3	(.01)	0.5	(.02)
	**Colombia**	*−0.0*	*(.05)*	0.8	(.09)	*−0.4*	*(.05)*	0.0	(.09)
	**Cuba**	0.3	(.22)	0.6	(.20)	0.2	(.25)	0.3	(.12)
	**Greece**	1.8	(.14)	2.5	(.19)	0.1	(.05)	1.5	(.13)
	**India**	0.4	(.01)	1.0	(.02)	*−0.1*	*(.01)*	1.1	(.05)
	**Indonesia**	0.5	(.03)	1.6	(.06)	0.1	(.02)	1.1	(.03)
	**Iran**	0.3	(.22)	0.3	(.34)	0.2	(.08)	0.9	(.18)
	**Malaysia**	1.0	(.02)	1.5	(.04)	0.5	(.02)	1.2	(.04)
	**Nepal**	0.4	(.06)	5.1	(.69)	0.2	(.02)	3.2	(.18)
	**Pakistan**	0.4	(.02)	0.8	(.04)	0.3	(.03)	1.4	(.02)
	**Philippines**	*−0.1*	*(.25)*	0.3	(.44)	*−0.3*	*(.25)*	0.7	(.23)
	**Portugal**	1.2	(.06)	1.6	(.08)	0.4	(.03)	1.4	(.04)
	**Rep. Korea**	0.8	(.03)	0.9	(.03)	0.2	(.02)	0.8	(.01)
	**Singapore**	0.5	(.08)	0.5	(.08)	*−0.5*	*(.10)*	0.2	(.05)
	**Spain**	0.8	(.03)	0.9	(.04)	0.3	(.03)	0.8	(.04)
	**Sri Lanka**	0.2	(.05)	0.6	(.10)	*−0.1*	*(.03)*	0.9	(.09)
	**Thailand**	0.8	(.02)	1.3	(.04)	0.3	(.03)	1.4	(.03)
	**Turkey**	0.9	(.09)	1.9	(.28)	*−0.2*	*(.04)*	1.3	(.04)
	**Venezuela**	0.4	(.13)	0.4	(.23)	0.4	(.08)	*−0.8*	*(.14)*
**Mature**	**Australia**	1.0	(.05)	1.3	(.07)	0.2	(.06)	0.6	(.06)
	**Austria**	0.2	(.03)	0.3	(.04)	*−0.1*	*(.06)*	0.2	(.04)
	**Belgium and Luxembourg**	*−0.1*	*(.06)*	*−0.2*	*(.07)*	0.2	(.05)	*−0.4*	*(.05)*
	**Canada**	*−0.0*	*(.08)*	*−0.1*	*(.10)*	0.0	(.08)	0.0	(.04)
	**Denmark**	0.0	(.05)	0.1	(.07)	*−0.1*	*(.05)*	*−0.3*	*(.07)*
	**Finland**	0.1	(.06)	0.0	(.07)	0.1	(.06)	0.2	(.06)
	**France**	*−0.1*	*(.05)*	*−0.2*	*(.06)*	0.1	(.06)	*−0.7*	*(.06)*
	**Germany**	*−0.4*	*(.04)*	*−0.4*	*(.04)*	*−0.4*	*(.06)*	*−0.5*	*(.05)*
	**Ireland**	0.2	(.02)	0.4	(.04)	*−0.0*	*(.02)*	0.3	(.02)
	**Italy**	0.2	(.03)	0.4	(.04)	*−0.1*	*(.03)*	0.4	(.02)
	**Japan**	*−0.1*	*(.05)*	*−0.1*	*(.05)*	*−0.1*	*(.04)*	0.3	(.03)
	**Netherlands**	*−0.2*	*(.09)*	*−0.2*	*(.10)*	0.0	(.09)	*−0.1*	*(.06)*
	**New Zealand**	0.2	(.07)	0.6	(.19)	0.0	(.08)	1.1	(.11)
	**Sweden**	0.1	(.08)	0.0	(.11)	0.2	(.06)	*−1.1*	*(.09)*
	**United Kingdom**	*−0.2*	*(.05)*	*−0.2*	*(.06)*	0.0	(.02)	*−0.3*	*(.03)*
	**USA**	0.0	(.04)	0.1	(.05)	*−0.2*	*(.05)*	*−0.1*	*(.03)*
	**World**	0.3	(.03)	0.5	(.04)	*−0.1*	*(.02)*	0.1	(.04)

Coupling coefficients of income and material consumption given by the income elasticity *b* in Eq. 1. Negative coefficients are shown in italics. The standard error of the coefficient is given in ().

We find that mature countries generally have lower economic-material coupling coefficients than developing countries, and sometimes even have significant negative coefficients, indicating absolute decoupling, in particular Germany, the Netherlands and the United Kingdom. Absolute decoupling can have complex and diverse causes: the recent dematerialization of the UK is mostly due to the decline of the manufacturing and construction sectors [52], with much of these activities being displaced overseas, as evidenced by the growth in its consumption-based emissions [53]. In contrast, in Germany, dematerialization is due to a combination of changes in energy composition, the decline in the use of construction minerals, and the collapse of polluting industries in the former GDR [54] – moreover, Germany is one of few Annex B countries with declining consumption-based emissions [28]. Interestingly, low economic-material coupling can also be seen in some of the developing economies, such as Colombia and the Philippines which in the case of Colombia was enabled by a significant increase in fossil fuel exports which drive GDP growth but don't have a large signal in DMC. This may also be explained by a high share of biomass in DMC, high population growth and declining biomass use per capita. Curiously, fossil exporter Venezuela is the only developing or emerging country with a large negative coupling between income and carbon emissions. In fact, over the period of our study, Venezuelan incomes declined, as did material consumption (Figure 1), while carbon emissions continued to rise.

Importantly, the group of successful developers constituted of Greece, Portugal, Republic of Korea, Singapore and Spain (Table 1) is characterized by high levels of economic-material coupling, with *b* above 0.5 for total DMC/cap, which is a level only exceeded by Australia in the mature group of countries. Similarly, with the notable exception of Colombia, the countries which went from developing to intermediate in Table 1, China, Indonesia, Thailand, also have material-economic coefficients above 0.5 i.e. considerable coupling of economic activity and resource use.

It would be mistaken, however, to conclude that a high material-economic coupling is a guaranteed recipe for successful development. Algeria, Brazil, Malaysia, and Turkey did not attain fully developed status in the 35 years covered by our time series, despite having consistently high levels of economic-material coupling. However, these countries started out with incomes below $3,000 per-capita, whereas the successful developers, with the exception of the Republic of Korea, started out with incomes above that level, so it may be a question of having sufficient time and growth rates to catch up with the mature industrialized nations.

The different types of materials show different levels of coupling with economic growth: in general the coupling is largest for minerals and fossils, followed by carbon emissions and total materials, with biomass having by far the lowest coupling, corroborating findings from previous studies, which have shown that biomass use is not related to economic development but much more to population trends, especially for mature economies [7], [8], [10], [41]. The levels and direction of material-economic coupling are by no means consistent across the board for the different types of materials considered. Most countries in the developing group have positive material-economic coupling for all the material categories, including fossil fuels represented by carbon emissions. In contrast, Colombia and Singapore have significantly decoupled their economic growth from biomass while increasing their reliance on minerals and fossils. Among the developed countries, Belgium and Luxembourg and Sweden have positive material-economic coupling for biomass (but still very low with 0.2) because of higher rates of timber harvest and high significance of wood processing industries, but negative for fossil fuels, whereas Italy and Ireland have weakly negative coupling for biomass, but positive for minerals and fossil fuels. The highest material-economic coupling of total DMC and in particular minerals and fossils in the mature group can be seen for Australia, New Zealand and Ireland, three countries with high levels of material use (Figure 1) and large export-oriented primary sectors in the case of Australia [55] and New Zealand.

What emerges from this analysis is a strongly differentiated picture of material and economic growth, showcasing the diversity of industrialization, materialization and development trajectories; in terms of growth rates, material-economic coupling and material composition changes. Although broad differences and trends between the developing and mature countries can be observed, such as higher economic-material coupling coefficients in the developing group, and higher coupling for minerals and fossils rather than biomass, these are far from universal. This analysis indicates the importance of carefully understanding the history and circumstances (such as natural resource endowments) of each country. To be able to identify some general patterns we employ panel analysis.

### Panel analysis

The first step in our panel analysis is to test the data for stationarity and co-integration. These tests are important in determining the type of panel analysis to conduct, and indicate whether there are in fact relations between material consumption and CO_2_ emissions and GDP.

The mixed results of the stationary tests (see File S1) confirm the heterogeneity of the time series data, since the development and history of the countries vary substantially. However, these results are not mirrored in the cointegration tests (see File S1) since all tests – except for mineral and fossils consumption in mature countries – point to a rather strong cointegrating relationship between GDP and the dependent variables. These tests support our hypothesis of significant coupling between GDP and the environmental indicators material use and CO_2_ emissions.

From a methodological point of view, we can thus proceed with panel estimations as presented in Eq. 2, accounting for heteroscedasticity by a weighted panel regression. We present estimations both with and without a time trend. The results are summarized in Table 3.

**Table 3:** Panel analysis results.

		Income		Quadratic income		Time trend		R^2^ adjusted	Peak income
Sample	Dependent variable	*b*	T-stat	*c*	T-stat	*e*	T-stat		(Eq. 3)
**All countries**	**Total DMC/cap**	0.81	12.78	0.09	8.72	−0.017	−5.97	0.997	*$65*
	**Total DMC/cap**	0.77	11.38	0.09	7.23			0.996	*$77*
	**Mineral+Fossil DMC/cap**	1.21	12.46	0.04	2.09	−0.022	−5.79	0.994	*$0*
	**Mineral+Fossil DMC/cap**	1.21	10.23	*0.03*	*0.93*			0.993	*$0*
	**Biomass DMC/cap**	0.25	6.42	*0.01*	*1.59*	−0.007	−7.09	0.996	*$0*
	**Biomass DMC/cap**	0.15	4.11	*0.01*	*1.32*			0.996	*$9*
	**CO2/cap**	0.73	11.09	*0.00*	*−0.03*	−0.013	−2.53	0.999	*NaN*
	**CO2/cap**	0.64	10.22	*−0.01*	*−1.04*			0.999	*1.33E+13*
**Emerging/**	**Total DMC/cap**	0.58	9.39	0.10	6.36	−0.006	−2.26	0.995	*$137*
**Developing**	**Total DMC/cap**	0.53	9.40	0.10	6.26			0.994	*$170*
	**Mineral+Fossil DMC/cap**	1.01	9.59	0.06	2.11	*−0.008*	*−1.73*	0.990	*$1*
	**Mineral+Fossil DMC/cap**	0.92	13.77	0.05	2.00			0.990	*$1*
	**Biomass DMC/cap**	0.23	5.94	*0.02*	*1.51*	−0.006	−4.47	0.996	*$11*
	**Biomass DMC/cap**	0.17	4.50	*0.03*	*1.95*			0.996	*$132*
	**CO2/cap**	0.71	12.21	*0.01*	*0.69*	*−0.003*	*−0.88*	0.998	*$0*
	**CO2/cap**	0.69	13.76	*0.01*	*0.56*			0.998	*$0*
**Mature**	**Total DMC/cap**	1.05	10.63	*−0.02*	*−0.11*	−0.022	−7.76	0.989	*1.70E+14*
	**Total DMC/cap**	1.04	10.04	*−0.34*	*−1.56*			0.988	*$126,108*
	**Mineral+Fossil DMC/cap**	1.41	11.16	*0.14*	*0.61*	−0.029	−6.64	0.982	*$202*
	**Mineral+Fossil DMC/cap**	1.38	10.53	*−0.32*	*−1.13*			0.981	*$232,634*
	**Biomass DMC/cap**	0.19	2.70	*−0.03*	*−0.32*	−0.005	−2.85	0.995	*$862,582*
	**Biomass DMC/cap**	*0.00*	*−0.02*	*−0.04*	*−0.50*			0.994	*$26,636*
	**CO2/cap**	0.67	5.45	−0.54	−3.40	−0.009	−2.47	0.992	$49,449
	**CO2/cap**	0.57	5.41	−0.78	−5.61			0.992	$38,688

Panel analysis results showing the coefficient values (as defined in Eq. 2), corresponding T-statistic and goodness-of-fit R^2^. Insignificant values are shown in italics.

The most striking result of the panel analysis is the overall lack of significance of the quadratic income (EKC) coefficients *c* – and when these are significant, they are most often positive (hence the very low “peak incomes”, which in those cases are really “valley incomes”), indicating a steadily increasing curve rather than an inverted U. Indeed, the only resource use category which exhibits a definite EKC-like behavior are the CO_2_ emissions of mature countries, although this may be due to displacement of carbon-intensive processes [25], [26]. However, the peak incomes, as derived from Eq. 3, are on the very high end of the sample's income spectrum, indicating that only the upward half of the inverted U is observed, with no observed decline in CO_2_ emissions at higher incomes. Altogether these results show no indication of lower resource use at higher or the highest incomes, quite the contrary: at the most a saturation or stabilization effect could be inferred for carbon emissions. This result should not be surprising given the central importance of fossil-fuels to modern economies.

The linear income coefficient *b*, in contrast, is the most significant in Table 3. This coefficient indicates the overall economic-material coupling in the sample, and can thus be compared to the values in Table 2 for individual countries. As in Table 2, the highest values are for mineral and fossil materials, followed by total materials and carbon emissions, with the lowest coupling found for biomass. However, there are important differences between country groups.

Surprisingly, the income-material coupling coefficient for minerals and fossils measured through panel analysis in Table 3 is significantly larger for mature economies (1.41 and 1.38) than for emerging and developing economies (1.01 and 0.92, with and without time trend respectively). This result is in marked contradiction with the long-term coupling values found for individual countries in Table 2. To explain this, the panel analysis coefficient has to be interpreted as a short term coupling value, indicating that fluctuations in economic growth rates have an immediate effect on material use: taken together, these values mean that mature industrialized countries have lower long-term economic-material coupling than developing or emerging economies, but that their economies are more tightly coupled to mineral and fossil resources in the short term: during economic recessions or booms, for example.

This is a highly relevant result, since it quantifies the material resilience of different types of economies over different time scales. Mature countries, with high per capita levels of mineral and fossil use, are less resilient to economic-material shocks in the short term (higher coupling indicating higher interdependency), but more resilient in the long term (lower coupling indicating lower dependency); whereas developing and emerging economies are most vulnerable in the long term, but exhibit more short-term resilience (lower dependency). There could be several explanations for this phenomenon: for mature economies, for instance the importance of the construction sector (responsible for 30–50% of DMC in industrial economies, and a known driver of material use [56], [57]) in economic cycles may lead to strong short term dependencies, while their highly developed physical infrastructure (roads, buildings, other forms of physical capital) enables a long term relative decoupling of materials use and economy. For developing and emerging economies, in contrast, long term economic growth is more clearly dependent on the continuous development of physical infrastructure and capital, leading to higher long-term coupling. Another factor could be the type of economic system (free-market or more politically controlled) of the two country groups. These explanations, while plausible, are only conjectures, and should be verified using complementary analysis in future work.

Trade and globalization may also play a role in these results and their interpretation. If the materials and carbon embodied in traded goods and services were taken into account, two outcomes would be possible. According to the convergence theory, the differences in couplings and timescales would be smoothed over, leading to globally uniform economic-material relationships. According to the global division of labor theory, these distinctions would not disappear altogether, or in some cases even be accentuated.

The reversal in short/long term coupling is not seen for fossil fuels alone, indicated by carbon emissions: in this case of carbon emissions, the economic coupling coefficient is lower for mature industrialized countries (0.67 and 0.57) than for developing/emerging countries (0.71 and 0.69), although the different coupling values in this case are not as far apart as for the mineral and fossil materials.

Innovatively, in this panel analysis, we tested the data for dependency on time alone, separate from economic growth trends (coefficient *e* in Eq. 2 and Table 3). In contrast with the quadratic income coefficient *c*, this term is always negative, and almost always significant, implying that material use per capita tends to decrease over time, once income effects have been taken into account. The time trend is not significant for developing and emerging countries, except for biomass and total materials, but it is significant for all material categories as well as carbon emissions for the whole sample and for mature economies.

The time trend can be interpreted as the short-term time-dependent (and income-independent) rate of improvement of material and carbon efficiency, related to technical improvements rather than economic growth. The value of the time trend coefficient, in fact, sets a short-term condition for absolute dematerialization: absolute per capita dematerialization can only occur if the growth rate of GDP per capita is smaller than –*e* / *b* (minus the time trend coefficient divided by the income coefficient). In the absence of the time trend, the condition for absolute dematerialization would be *b* < 0 for any positive economic growth rate, so this could be seen as an improvement: the existence of a time trend allows for some positive economic growth. This understanding of the time trend is confirmed by the fact that the positive income coupling coefficient is always smaller in the absence of the time trend, indicating that the time-related effect mitigates the coupling between economic growth and increases in material use. The limit on per capita economic growth for dematerialization comes to 2.1% for total materials and to 1.8% for carbon dioxide, for the whole sample. Interestingly, these rates are above the long term global average of 1.5% per capita GDP growth. This paradox (no past dematerialization or decarbonisation despite growth rates below the “upper limit”) is again due to the short-term nature of the panel analysis results, indicating that the time trend and resulting upper limit to economic growth compatible with dematerialization/decarbonisation can only be interpreted as a short term effect.

## General Discussion

Taken together, our results are consistent with our hypothesis that there exist significant differences in the economic dependency for environmental resources between mature industrialized countries and their emerging or developing counterparts, despite the great heterogeneity in material and economic development in each group. The different analytical approaches employed provide complementary insights into the complexity of the relation between economic growth and resource use. In particular, the expected result of higher material-economic coupling measured in the developing/emerging country trajectories, contrasted with the higher coupling measured for mature countries in the panel analysis, show that the scale of time dependency shifts the result, with the first type of coupling representing longer-term dependency, and the second shorter-term. This type of distinction may become quite important in understanding the environmental implications of economic booms and crises, as well as longer term growth, and may have policy important implications for guiding economic development towards lower resource and emissions intensity.

The implication and meaning of the highly significant time trend seen in the panel analysis are extremely interesting. The time trend can be understood as a short-term but steady shift in the coupling of economic development and resource use over time, corresponding to a shift in the overall income elasticity of the relation (see for instance [58]). This type of shift over time has been seen in life expectancy vs. income [59] and human development vs. energy and carbon emissions [60], and is generally interpreted as an improvement in the international efficiency of producing desirable outcomes (economic or social) for a given level of economic or material wealth.

The time trend may thus be interpreted as the “autonomous technological progress” mirroring the constant improvement in material productivity. However, as the current analysis shows, increased material efficiency is by no means sufficient for absolute decoupling between the economy and environmental resources. Technological progress leads to economic growth, and might not only be used to reduce resource consumption but also for exploiting resources more intensively or cost-effectively. Indeed, recent research into the role physical and technical factors of production in economic growth has shown that technical efficiency is a major factor of production, and can account for most of the Solow Residual of traditional growth models [61]. The Solow Residual, also known as Total Factor Productivity, measures the gap between real growth and growth predicted based on increases in labor and capital, and has often been interpreted as the contribution of innovation. The role of technical progress in driving economic growth may explain why, when we derive a maximum income growth rate consistent with absolute dematerialization or decarbonization, this income growth rate is below historic growth rates.

## Conclusion and Next Steps

This study has established some key findings, and raised new questions which need to be further explored by future research. Key findings include that developing and emerging countries indeed have a higher long term economic dependency on materials and fossil fuels than mature economies, but the short term economic-material coupling is paradoxically higher for mature industrialized countries. EKC-like behavior, indicating a slowing down of environmental resource use at higher incomes, is only seen for the carbon emissions of the mature economies in our sample, and we observe nothing consistent with an actual decline at higher incomes. The idea to grow first and to deal with environmental issues later has been proven false empirically. Its appeal was and is based more in wishful thinking rather than sound evidence.

For the first time, we observe a strong and significant negative time trend, independent of income, for the total sample of countries and all material categories and carbon emissions. We can interpret this as a rate of “autonomous technical progress,” and it would be interesting to test this on other data sets and with other complementary variables. If this time trend effect is confirmed, and measurable as a long-term as well as short-term effect, the finding would be extremely significant, since it would allow to effectively set the pace for economic growth consistent with absolute decarbonization and dematerialization. How does this rate change if different forms of GDP (for instance Purchasing Power Parity), or consumption-based material [27] and carbon accounts [26] were used thereby acknowledging the global characteristics of trade flows among countries? Do consumption-based accounting measures diminish the differences observed between developing and industrialized economies? If we were to divide up the analysis into different time periods, would we observe systematic trends in economic-material coupling and the autonomous technical progress rates? It may also be possible to conduct a parallel analysis on the material and carbon intensities of these economies, which would yield complementary insights.

A number of policy relevant conclusions can be drawn without any further research. The most important is that current modes of development, both for emerging and already industrialized economies, are fundamentally unsustainable. There is no empirical evidence for decarbonization or dematerialization at higher economic growth rates or incomes. Global environmental sustainability thus requires a fundamental shift away from industrial development as usual. Recent initiatives focusing on alternative forms of economic growth by new investment or changing existing investment pathways in favor of a green economy, i.e. such economic activities that delivery goods and services such as housing, mobility and food at a much lower resource and emissions intensity [1], or challenging growth altogether and prioritizing human well-being and non-resource-intensive development [2], are thus necessary first steps towards the radical change required to allow nine billion people to attain high human development within planetary environmental limits [60]. These initiatives translate into different policies for mature industrialized countries, which must curb their consumption while preserving and enhancing high living standards, and developing and emerging countries, which should avoid the trap of resource-intensive development. The international focus on human well-being, rather than economic activity, as a development priority, evidenced by the April 2012 “Happiness and Well-being: Defining a New Economic Paradigm” United Nations conference in New York, demonstrates that this necessity is starting to be acknowledged at the highest level, although its translation into practical policies is likely to be quite challenging.

## Supporting Information

File S1 **Data, methodological details, and results. Table S1, References for country material flow sources. Table S2, Individual country trajectories. Table S3, Panel stationarity tests of explanatory and dependent variables (H_0_: non-stationarity). Table S4, Panel cointegration tests.**
(DOCX)
