# Supplementary material for: Development and Dematerialization: An International Study
Source: PLoS One. 2013 Oct 21;8(10):e70385. doi: 10.1371/journal.pone.0070385 (PMC3804739; doi:10.1371/journal.pone.0070385)
Supplement: File S1 — Data, methodological details, and results. Table S1, References for country material flow sources. Table S2, Individual country trajectories. Table S3, Panel stationarity tests of explanatory and dependent variables (H0: non-stationarity). Table S4, Panel cointegration tests. (DOCX) [file pone.0070385.s001.docx]

Development and dematerialization: an international study: Supplementary Materials

Julia K. Steinberger (1,2), Fridolin Krausmann (1), Michael Getzner (3), Heinz Schandl (4), Jim West (4)

Affiliations

1. Institute of Social Ecology, Vienna, Alpen Adria Universitaet Klagefurt-Wien-Graz, Schottenfeldgasse 29, 1070 Vienna, Austria. [julias@alum.mit.edu](mailto:julias@alum.mit.edu), [fridolin.krausmann@aau.at](mailto:fridolin.krausmann@aau.at)
2. Sustainability Research Institute, School of Earth & Environment, University of Leeds, Leeds LS2 9JT, United Kingdom, [julias@alum.mit.edu](mailto:julias@alum.mit.edu)
3. Center of Public Finance and Infrastructure Policy, Vienna University of Technology, Karlsplatz 13, 1040 Vienna, Austria, [michael.getzner@tuwien.ac.at](mailto:michael.getzner@tuwien.ac.at)
4. CSIRO Ecosystem Sciences, Clunies Ross St, Black Mountain, ACT 2601, Australia, [Heinz.Schandl@csiro.au](mailto:Heinz.Schandl@csiro.au) , [Jim.West@csiro.au](mailto:Jim.West@csiro.au) .

# Data

Table S1: references for country material flow sources (details of references are listed at the end of the Supplementary Materials).

| **Country** | **Material Flow Analysis data source** | **Years covered** |
| --- | --- | --- |
| Algeria | SEC 2011 | 1970-2005 |
| Argentinia | SEC 2011 | 1970-2004 |
| Australia | Schandl and West 2010 | 1970-2005 |
| Austria | Weisz 2006 | 1970-2004 |
| Bangladesh | Schandl and West 2010 | 1970-2005 |
| Belgium+Luxembourg | Weisz et al. 2006 | 1970-2004 |
| Brazil | Mayer 2010 | 1970-2005 |
| Canada | SEC 2011 | 1980-2003 |
| China | Schandl and West 2010 | 1970-2005 |
| Colombia | SEC 2011 | 1970-2005 |
| Cuba | Eisenhut 2009 | 1970-2004 |
| Denmark | Weisz et al. 2006 | 1970-2004 |
| Finland | Weisz et al. 2006 | 1970-2004 |
| France | Weisz et al. 2006 | 1970-2004 |
| Germany | Weisz et al. 2006 | 1970-2004 |
| Greece | Weisz et al. 2006 | 1970-2004 |
| India | Singh et al. 2012 | 1970-2005 |
| Indonesia | Schandl and West 2010 | 1970-2005 |
| Iran | Schandl and West 2010 | 1970-2005 |
| Ireland | Weisz et al. 2006 | 1970-2004 |
| Italy | Weisz et al. 2006 | 1970-2004 |
| Japan | Krausmann et al. 2011 | 1970-2005 |
| Malaysia | Schandl and West 2010 | 1970-2005 |
| Nepal | Schandl and West 2010 | 1970-2005 |
| Netherlands | Weisz et al. 2006 | 1970-2004 |
| New Zealand | Schandl and West 2010 | 1970-2005 |
| Pakistan | Schandl and West 2010 | 1970-2005 |
| Philippines | Schandl and West 2010 | 1970-2005 |
| Portugal | Weisz et al. 2006 | 1970-2004 |
| Rep. Korea | Schandl and West 2010 | 1970-2005 |
| Singapore | Schandl and West 2010 | 1970-2005 |
| Spain | Weisz et al. 2006 | 1970-2004 |
| Sri Lanka | Schandl and West 2010 | 1970-2005 |
| Sweden | Weisz et al. 2006 | 1970-2004 |
| Thailand | Schandl and West 2010 | 1970-2005 |
| Turkey | Schandl and West 2010 | 1970-2005 |
| United Kingdom | Weisz et al. 2006 | 1970-2004 |
| USA | Gierlinger and Krausmann 2012 | 1970-2005 |
| Venezuela | SEC 2011 | 1970-2005 |
| World | Krausmann et al. 2009 | 1970-2005 |

# Methodological details

We present below some details regarding the quantitative methodologies summarized in the main article.

## Cluster analysis

The cluster analysis is conducted using the Matlab software language “linkage” and “cluster” functions, based on the average linkage between all pairs of objects in any two clusters, and standardized Euclidean distances, which are inversely weighted by the sample variance in that coordinate, in order to make the dimensions unitless and cover the same range. The number of clusters is set to 8, to achieve a sufficient level of differentiation within the groups (otherwise, the clusters tend to be formed of 1 or 2 exceptional countries, like Finland and Australia, and all the others).

# Results

## Cluster analysis results

The cluster analysis is conducted at two points in time: at the beginning and end of the time span of our data, 1970 and 2004. The cluster analysis considers both economic (GDP/cap, logged to take into account the two orders of magnitude range) and physical variables (DMC/cap). Because we are interested in development trajectories as well as development status, these variables are considered in terms of their static and growth values (the growth values are averaged between 1970-1975 and 2000-2004 in order to smooth over exceptionally variable years). The country clusters are thus defined in terms of their dynamic growth rates, as well as their material and economic status.

### Results of the 1970 clustering

The results of the 1970 levels, growth rates averaged between 1970-1975 are shown below (the number in parentheses is the number of countries in a given cluster). Clusters 4 & 5 comprise the most developed countries, next come intermediate clusters 1 & 2, with countries which have lower levels of development, but faster growth. Cuba, in cluster 3, is distinguished from this intermediate group by its material degrowth, while Algeria, in cluster 8, it characterised by extreme material growth. Then comes cluster 6, with the developing countries, from which Bangladesh in cluster 7 is separated because of its economic degrowth during that time period. Interestingly, contrary to our initial expectation, the highest growth rates are seen for the intermediate countries, while the developing countries have growth rates which are similar to those of the most mature countries.

Cluster 1 (4): Greece, Portugal, Spain, Turkey;

Cluster 2 (5): Brazil, Iran, Malaysia, Rep. Korea, Singapore;

Cluster 3 (1): Cuba;

Cluster 4 (16): Argentina, Austria, Australia, Belgium+Luxembourg, Germany, Denmark, France, Ireland, Italy, Japan, Netherlands, New Zealand, Sweden, Venezuela, United Kingdom, USA;

Cluster 5 (1): Finland;

Cluster 6 (9): China, Colombia, India, Indonesia, Nepal, Philippines, Pakistan, Sri Lanka, Thailand;

Cluster 7 (1): Bangladesh;

Cluster 8 (1): Algeria


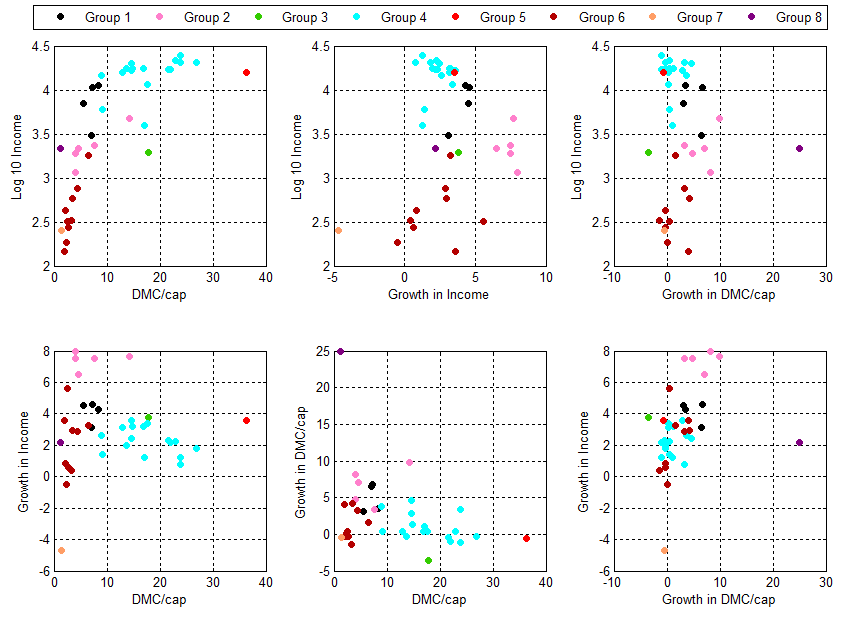


### Results for 2004 clustering

The results of the 2004 levels, growth rates averaged between 2000-2004 are shown below (the number in parentheses is the number of countries in a given cluster). Clusters 2 & 5 comprise the most developed countries, which have been augmented by Greece, Portugal, Spain and Rep. Korea. Singapore, in cluster 8, belongs to this group, and is distinguished by its large material degrowth, due to the end of large construction phase in the city-state (see Schulz ([Schulz, 2007](#_ENREF_2)) for the details on the history of Singapore’s material flows). The intermediate group, formed by clusters 1, 3, 6 & 7, has a significantly changed membership: it has been augmented by Argentina and Venezuela (moving down from their mature status in 1970) as well as China, Colombia, Indonesia and Thailand (moving up from the developing group). The developing group of cluster 4 has thus shrunk. Cuba is distinguished from the other intermediate countries by an exceptional combination of material degrowth and economic growth, whereas China is in a cluster of its own due to its material and economic growth.

Cluster 1 (5): Argentina, Brazil, Colombia, Turkey, Venezuela;

Cluster 2 (5): Algeria, Indonesia, Iran, Malaysia, Thailand;

Cluster 3 (6): Bangladesh, India, Nepal, Pakistan, Philippines, Sri Lanka;

Cluster 4 (2): Australia, Finland;

Cluster 5 (17): Austria, Belgium+Luxembourg, Germany, Denmark, France, Greece, Ireland, Italy, Japan, Rep. Korea, Netherlands, New Zealand, Portugal, Spain, Sweden, United Kingdom, USA;

Cluster 6 (1): Cuba;

Cluster 7 (1): China;

Cluster 8 (1): Singapore.


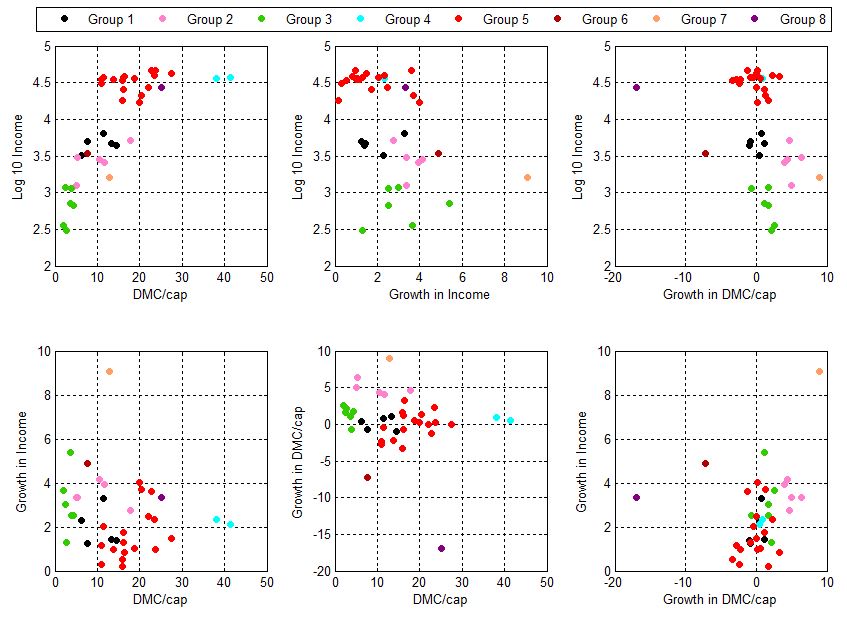


Note: material flow data for Canada is only available for 1980-2003, but Canada is straightforwardly classified as a mature industrialized country.

## Individual country trajectories

Table S2:

|  | Population | GDP/cap | Total DMC/cap | Mineral DMC/cap | Biomass DMC/cap | CO2/cap |
| --- | --- | --- | --- | --- | --- | --- |
| Units | 1000 pers | USD | tons/cap | tons/cap | tons/cap | tons CO2/cap |
| Year | 2005 | 2005 | 2005 (*) | 2005 (*) | 2005 (*) | 2005 |
| Algeria | 32,900 | 3,142 | 5.4 | 4.6 | 0.8 | 4.2 |
| Argentina | 38,700 | 4,730 | 14.5 | 4.1 | 10.4 | 4.1 |
| Bangladesh | 153,100 | 376 | 2.0 | 0.8 | 1.2 | 0.3 |
| Brazil | 186,100 | 4,740 | 13.3 | 4.6 | 8.7 | 1.9 |
| China | 1,303,700 | 1,766 | 12.8 | 10.3 | 2.4 | 4.3 |
| Colombia | 43,000 | 3,358 | 6.3 | 2.2 | 4.1 | 1.4 |
| Cuba | 11,200 | 3,810 | 7.6 | 2.3 | 5.3 | 2.2 |
| Greece | 11,100 | 21,819 | 20.3 | 16.9 | 3.5 | 8.9 |
| India | 1,094,600 | 768 | 3.7 | 2.1 | 1.7 | 1.3 |
| Indonesia | 219,200 | 1,304 | 5.1 | 2.5 | 2.5 | 1.6 |
| Iran | 69,100 | 2,976 | 10.5 | 7.1 | 3.5 | 6.2 |
| Malaysia | 25,600 | 5,382 | 17.8 | 10.0 | 7.8 | 7.2 |
| Nepal | 27,200 | 303 | 2.6 | 0.2 | 2.5 | 0.1 |
| Pakistan | 155,800 | 701 | 4.4 | 1.4 | 2.9 | 0.9 |
| Philippines | 85,500 | 1,156 | 3.8 | 1.5 | 2.3 | 0.9 |
| Portugal | 10,500 | 18,122 | 16.0 | 12.0 | 4.0 | 6.2 |
| Rep. Korea | 48,100 | 17,551 | 19.9 | 18.5 | 1.4 | 9.6 |
| Singapore | 4,300 | 28,423 | 25.1 | 24.9 | 0.3 | 14.0 |
| Spain | 43,400 | 26,042 | 16.2 | 12.6 | 3.7 | 8.1 |
| Sri Lanka | 19,700 | 1,241 | 2.5 | 1.1 | 1.3 | 0.6 |
| Thailand | 65,900 | 2,674 | 11.6 | 7.4 | 4.2 | 4.1 |
| Turkey | 71,200 | 6,786 | 11.3 | 7.8 | 3.5 | 3.3 |
| Venezuela | 26,600 | 5,428 | 8.6 | 5.0 | 3.6 | 6.0 |
| Australia | 20,400 | 37,474 | 41.3 | 31.5 | 9.9 | 17.8 |
| Austria | 8,200 | 36,792 | 18.6 | 14.4 | 4.2 | 8.8 |
| Belgium+Luxembourg | 10,900 | 37,855 | 16.1 | 11.8 | 4.3 | 10.9 |
| Canada | 32,300 | 35,088 | 32.3 | 24.2 | 8.1 | 17.3 |
| Denmark | 5,400 | 47,577 | 23.8 | 17.0 | 6.8 | 8.6 |
| Finland | 5,200 | 37,290 | 38.1 | 29.0 | 9.1 | 10.4 |
| France | 60,900 | 35,262 | 13.9 | 8.7 | 5.1 | 6.4 |
| Germany | 82,500 | 33,811 | 15.8 | 12.5 | 3.3 | 9.8 |
| Ireland | 4,200 | 48,533 | 22.8 | 13.7 | 9.1 | 10.4 |
| Italy | 58,600 | 30,332 | 10.9 | 8.1 | 2.8 | 8.0 |
| Japan | 127,800 | 35,627 | 11.1 | 9.8 | 1.3 | 9.7 |
| Netherlands | 16,300 | 39,122 | 16.3 | 13.3 | 3.0 | 10.6 |
| New Zealand | 4,100 | 27,298 | 21.9 | 7.4 | 14.5 | 8.1 |
| Sweden | 9,000 | 41,066 | 23.4 | 15.6 | 7.7 | 5.7 |
| United Kingdom | 60,200 | 37,859 | 11.5 | 8.9 | 2.6 | 9.0 |
| USA | 295,600 | 42,562 | 27.5 | 21.7 | 5.8 | 19.7 |
| World | 6,466,800 | 7,070 | 8.9 | 6.0 | 2.9 | 4.5 |

(*) 2004 values for EU-15, Argentina, Cuba, 2003 for Canada.

## Panel analysis

Table S3 shows that results are mixed for all country groups and variables, depending on the assumptions about common vs. individual unit root processes. GDP is non-stationary in levels throughout the sample, pointing to steady economic growth in the long run. Total DMC seems to be stationary for the total sample as well as for the mature countries while developing countries seem to follow a steady growth in their material consumption. Minerals consumption is also rather stationary with some inconclusive results for mature countries. For biomass and CO2, we also find mainly stationary or rather inconclusive results.

Table S4 presents the results for the cointegration tests exploring relations between GDP on the one hand, and CO2 and material consumption on the other hand. Again, results are shown for different assumptions and test statistics.

Table S3: Panel stationarity tests of explanatory and dependent variables (H_0_: non-stationarity)

| **Variables -Stationarity Tests** | | H0: | Non-stationarity | |  |  |  |  |
| --- | --- | --- | --- | --- | --- | --- | --- | --- |
|  | Variables | Level |  |  |  | 1st difference | | Conclusion |
|  | Assumption | Common unit root process | Individual unit root process | | Common unit root process | Individual unit root process | | |
|  |  | Levin, Lin & Chu | ADF-Fisher | PP-Fisher | Levin, Lin & Chu | ADF-Fisher | PP-Fisher |  |
| YRPOP | World | -2.605*** | 71.321 | 73.935 | 14,956*** | 424,678*** | 476,662*** | I(1) |
|  | Industrialized | -3,43122*** | 25.5281 | 32.5771 | -10,2564*** | 151,098*** | 151,453*** | I(1) |
|  | Developing | -0.52838 | 45.7933 | 41.3581 | -10,9976*** | 273,580*** | 325,209*** | I(1) |
| DMC | World | -3,84168*** | 120,258*** | 130,082*** | -33,1085*** | 1162,61*** | 1472,72*** | I(0) |
|  | Industrialized | -2,51292*** | 70,5677*** | 74,8311*** | -22,0341*** | 455,429*** | 506,163*** | I(0) |
|  | Developing | -3,10088*** | 49.6906 | 55.251 | -24,8759*** | 707,182*** | 966,56*** | I(1) |
| DMC-minerals + fossils | World | -4,94123*** | 124,009*** | 111,433** | -29,8771*** | 1016,56*** | 1157,08*** | I(0) |
|  | Industrialized | -0.16712 | 51,2208** | 44,2533* | -18,8014*** | 362,514*** | 387,235*** | inconclusive, rather I(1) |
|  | Developing | -5,18715*** | 72,7883** | 67,1801** | -23,4241*** | 654,042*** | 769,84*** | I(0) |
| DMC-bio | World | -3,74098*** | 149,782*** | 153,987*** | -41,7207*** | 1771,17*** | 2999,17*** | I(0) |
|  | Industrialized | -3,81875*** | 86,2552*** | 91,8116*** | -28,6487*** | 803,717*** | 1708,64*** | I(0) |
|  | Developing | -2,46484*** | 63,527* | 62,1756* | -30,6565*** | 967,457*** | 1290,53*** | inconclusive, rather I(0) |
| CO2 | World | -4,64687*** | 101,581* | 112,41*** | -29,9823*** | 1102,27*** | 1398,06*** | inconclusive, rather I(0) |
|  | Industrialized | -2,83345*** | 44,6588* | 43,6644* | -24,2181*** | 552,647*** | 659,444*** | inconclusive, rather I(0) |
|  | Developing | -3,98519*** | 56,9224** | 68,7456** | -20,0663*** | 549,624*** | 738,618*** | I(0) |

Table S4: Panel cointegration tests (H_0_: no cointegration between GDP per capita and the dependent variables)

| **Cointegration tests of YRPOP and …** | | H0: | No cointegration | |  |  |  |  |
| --- | --- | --- | --- | --- | --- | --- | --- | --- |
|  | Assumption | Common AR coefficients | |  | Individual Ar coefficients | |  | Conclusion |
|  |  | Panel rho-Statistic | Panel PP-Statistic | Panel ADF-Statistic | Group rho-Statistic | Group PP-Statistic | Group ADF-Statistic |  |
| DMC | World | -6,202961*** | -5,969705*** | -6,212418*** | -4,593915*** | -5,805509*** | -5,873886*** | HA |
|  | Industrialized | -5,268056*** | -4,869351*** | -4,936479*** | -2,78316*** | -4,230388*** | -4,359523*** | HA |
|  | Developing | -4,49281*** | -4,355037*** | -4,530825*** | -3,658278*** | -4,040782*** | -4,023618*** | HA |
| DMC-minerals + fossils | World | -9,281431*** | -8,745356*** | -7,285038*** | -3,537668*** | -3,793903*** | -2,834667*** | HA |
|  | Industrialized | -5,715232*** | -0.474545 | -0.498886 | -2,550198*** | -1,701826** | -2,198584** | H0 |
|  | Developing | -7,2049*** | -8,114665*** | -6,773899*** | -2,484881*** | -3,508373*** | -1,864403** | HA |
| DMC-bio | World | -4,645479*** | -4,53602*** | -4,39759*** | -6,053045*** | -8,251414*** | -8,516939*** | HA |
|  | Industrialized | -6,419447*** | -4,730584*** | -5,350099*** | -5,216475*** | -7,187071*** | -6,94893*** | HA |
|  | Developing | -2,644052*** | -2,982923*** | -2,682776*** | -3,555214*** | -4,78431*** | -5,321543*** | HA |
| CO2 | World | -4,624471*** | -5,850175*** | -6,328748*** | -2,862971*** | -4,610696*** | -4,66452*** | HA |
|  | Industrialized | -4,892013*** | -4,561008*** | -4,581873*** | -2,695245*** | -3,655819*** | -3,657491*** | HA |
|  | Developing | -3,107185*** | -4,330359*** | -4,767756*** | -1,495421* | -2,967419*** | -3,035541*** | HA |

**References**

Eisenhut, S., 2009. Material Flow Analysis: Cuba. Masterthesis, Universität Wien.

Gierlinger, S., Krausmann, F., 2012. The physical economy of the United States of America: Extraction, trade and consumption of materials from 1870 to 2005. Journal of Industrial Ecology, 16 (3), 365-377.

Krausmann, F., Gingrich, S., Eisenmenger, N., Erb, K.-H., Haberl, H., Fischer-Kowalski, M., 2009. Growth in global materials use, GDP and population during the 20th century. Ecological Economics, 68 (10), 2696-2705.

Krausmann, F., Gingrich, S., Nourbakhch-Sabet, R., 2011. The metabolic transition in Japan: A material flow account for the period 1878 to 2005. Journal of Industrial Ecology, 15 (6), 877-892.

Mayer, A. Resource use and material flows in the Brazilian economy, 1960-2005. Social Ecology Working Paper 118, Vienna: Inst. of Social Ecology.

SEC 2011. Material Flow Database of the Institute of Social Ecology. Vienna: Institute of Social Ecology.

Schandl, H., West, J., 2010. Resource use and resource efficiency in the Asia-Pacific region. Global Environmental Change, 20 (4), 636-647.

Singh, S.J., Krausmann, F., Gingrich, S., Haberl, H., Erb, K.-H., Lanz, P., Martinez-Alier, J., Temper, L., 2012. India's biophysical economy, 1961 – 2008. Sustainability in a national and global context. Ecolgical Economics, 76, 60-69.

Weisz, H., Krausmann, F., Amann, C., Eisenmenger, N., Erb, K.-H., Hubacek, K., Fischer-Kowalski, M., 2006. The physical economy of the European Union: Cross-country comparison and determinants of material consumption. Ecological Economics, 58 (4), 676-698.
